# UniChem: extension of InChI-based compound mapping to salt, connectivity and stereochemistry layers

**DOI:** 10.1186/s13321-014-0043-5

**Published:** 2014-09-04

**Authors:** Jon Chambers, Mark Davies, Anna Gaulton, George Papadatos, Anne Hersey, John P Overington

**Affiliations:** ChEMBL, European Molecular Biology Laboratory – European Bioinformatics Institute (EMBL-EBI), Wellcome Trust Genome Campus, Cambridge, CB10 1SD Hinxton UK

**Keywords:** UniChem, Standard InChI, InChIKey, Chemical databases, Data integration, Connectivity search

## Abstract

UniChem is a low-maintenance, fast and freely available compound identifier mapping service, recently made available on the Internet. Until now, the criterion of molecular equivalence within UniChem has been on the basis of complete identity between Standard InChIs. However, a limitation of this approach is that stereoisomers, isotopes and salts of otherwise identical molecules are not considered as related. Here, we describe how we have exploited the layered structural representation of the Standard InChI to create new functionality within UniChem that integrates these related molecular forms. The service, called `Connectivity Search' allows molecules to be first matched on the basis of complete identity between the connectivity layer of their corresponding Standard InChIs, and the remaining layers then compared to highlight stereochemical and isotopic differences. Parsing of Standard InChI sub-layers permits mixtures and salts to also be included in this integration process. Implementation of these enhancements required simple modifications to the schema, loader and web application, but none of which have changed the original UniChem functionality or services. The scope of queries may be varied using a variety of easily configurable options, and the output is annotated to assist the user to filter, sort and understand the difference between query and retrieved structures. A RESTful web service output may be easily processed programmatically to allow developers to present the data in whatever form they believe their users will require, or to define their own level of molecular equivalence for their resource, albeit within the constraint of identical connectivity.

**Electronic supplementary material:**

The online version of this article (doi:10.1186/s13321-014-0043-5) contains supplementary material, which is available to authorized users.

## Background

The rapidly increasing number and diversity of small molecule-containing resources on the Internet presents an ongoing and time-consuming data integration challenge to those faced with data federation and maintaining links between equivalent chemical entities in these different resources. UniChem was developed as an automated, extensible, and scalable solution to this problem and was recently made publicly available [1]. Using the hashed version of the Standard InChI; the Standard InChIKey, as the normalization standard, UniChem is able to efficiently produce up to date mappings between small molecules in different resources on the basis of complete identity at the level of this widely adopted and stable standard [2]. Other resources provide similar mapping services [3-9], and some differences, advantages and disadvantages of these over UniChem have already been discussed [1].

However, for a variety of reasons, molecules that many scientists would consider equivalent in the context of their particular field (e.g. pharmacology, docking, etc.), are quite often depicted differently across different resources. Frequently, these depictions have different Standard InChIs and so cannot be integrated by simply matching on Standard InChIKey. The variety of such essentially similar structural forms that exist across chemistry web resources but which are not integrated by exact matching on Standard InChI is considerable (Hersey A, Chambers J, Bento P, Bellis L, Gaulton A, Overington JP: Chemical Databases: Curation or Integration by User-Defined Equivalence?, submitted). Curation errors may also account for some of these differences. Complex stereochemistry for example, can often be a challenge to extract and curate, although similar discrepancies also occur through simple differences of opinion on the true stereochemistry of a molecule. Some resources seek to extract and reproduce data from other sources, but aim to maintain any ambiguity that may have been present in the original depiction (such as the presence of undefined stereochemistry). In addition, isotopic forms of small molecules are considered equivalent in some contexts and different in others, although once again, all these forms will have different Standard InChIs. Lastly, molecules in different protonation states or present as different salt forms or within mixtures, will all yield different Standard InChIs, though for many purposes it is essential to be able to relate them to one another. In these respects, the original specification of UniChem (creating links only on the basis of Standard InChI identity) appears too narrow and constrained for some purposes. A less stringent criterion for producing mappings is therefore appropriate for some users.

The original developers of the InChI foresaw exactly this issue [2], and deliberately designed the InChI in such a way such that molecules could be compared on different levels of structural specification. Thus, progressively increasing levels of structural definition are encoded within consecutive `layers' of the InChI string, and separate components of a mixture or salt are represented as sub-layers, all in a simple parsable format. Furthermore, the initial layers which define molecular formula and atom connectivity and are codified separately in the First InChIKey Hash Block (FIKHB) of the InChIKey. The FIKHB alone can therefore be used as a simple way to compare molecules on the basis of atom connectivity, and has indeed been used successfully to interlink substances with the same skeleton [4,5], but not across mixtures and salts. Although other mechanisms for normalization and searching at different levels of structural representation exist [10], including across mixtures and salts [11], InChI was used in the current work because the existing UniChem application was originally built on this widely accepted standard, and many sources used by UniChem make this identifier easily available.

Here, we have exploited the features of the Standard InChI described above to provide new functionality within UniChem which enables mappings to be made between molecules that share common atom connectivity, even across mixtures and salts. An important early requirement for this service was that, as far as possible, and within the constraint of identity at the connectivity level, the user should be able to define for themselves their own criteria of molecular equivalence, since this may vary between users and areas of expertise. For this reason, querying options for refining the search were considered important. It was also decided that result sets should be fully annotated with structural differences, allowing users to either manually browse or process programmatically, and to apply, as far as possible, their own rules for molecular equivalence. To achieve this, it was recognized that the Standard InChI, and not simply the Standard InChIKey, assigned to the Query term would need to be compared to the Standard InChI assigned to the retrieved data. In this way, differences between the query and retrieved data could be annotated at the highly granular level of the separate Standard InChI layers. Lastly, a key requirement was that the service should be fast, so that like the original UniChem services, the new service could be used as an `on the fly' web service.

## Construction and content

### Database schema

**Figure 1:**
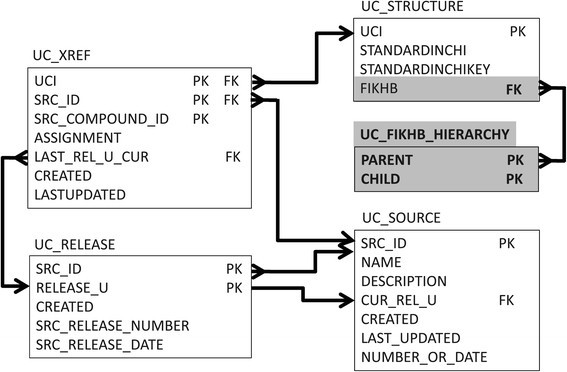
**Modifications to the UniChem schema required to implement connectivity search.** The UniChem schema (described previously [1]) was modified by the addition of the UC_FIKHB_HIERARCHY table and the FIKHB field within the UC_STRUCTURES table. Both additions are highlighted with bold and shading. Full details of the function of these additions are given in the text. For clarity, not all fields are shown. Primary/foreign key constraints are indicated by solid arrows. PK = Primary Key, FK = Foreign Key.

Since a major requirement was that the service should be fast, key design decisions were taken to optimize speed. It was recognized that probably the slowest part of Connectivity Search would be the multiple database lookups that would be required to retrieve components of multi-component Standard InChIs (i.e. mixtures and salts). Optimizing this lookup process was therefore identified as important, and would be greatly assisted by using the FIKHB instead of the Standard InChI Connectivity layer from which it derived, as its fixed short length would lend itself to being more efficiently queried than a longer variable length string in the setting of an indexed database field. For this reason, the FIKHB was used as the key to create lookups to and from the main structure table, with a separate table to define the mappings between composite and single component Standard InChI connectivity layers. To implement Connectivity Searching, the UniChem schema (originally consisting of four main tables, as described before [1]), was extended to include an additional table called UC_FIKHB_HIERARCHY, and an additional field within the UC_STRUCTURE table, as shown in Figure 1. The purpose of the simple 2 field table UC_FIKHB_HIERARCHY is to store the `parent-child' relationships between the FIKHBs of a multiple component Standard InChI and its corresponding single component Standard InChIs, and thus serves to permit queries which search within multi-component Standard InChIs (see criterion C, below). New records are added to this table at load time, but only when the loader detects a multiple component Standard InChI with a novel FIKHB: Thus multiple component Standard InChIs with the same connectivity as an existing Standard InChI in UniChem, but with novel stereochemistry, or isotopic composition, need not be parsed and inserted into this table, since the mapping between connectivity layers will already exist in this table. Single component Standard InChIs created from novel multi-component Standard InChIs during this process are not stored, although they may already exist in the UC_STRUCTURES table anyway. A composite primary key of both `PARENT' and `CHILD' fields ensures that the data in this table is non-redundant with respect to the connectivity mapping for a given composite Standard InChI.

The purpose of the additional field in the UC_STRUCTURE table, called FIKHB, is to store the FIKHB of the corresponding Standard InChIKey in the same record in the UC_STRUCTURE table, and is created from the STANDARDINCHIKEY field of this record at load time. These two changes; the addition of one table, and one field to an existing table, as shown in Figure 1, were the only changes required to implement Connectivity Search in the previously defined UniChem schema [1].

During querying, the pattern of lookups between the UC_STRUCTURE and UC_FIKHB_HIERARCHY tables is dependent upon the value of criteria C (described below). Thus, for example, queries requiring a search for the `single component InChIs of a multiple component InChI' require that first the `CHILD' FIKHBs of a query `PARENT' FIKHB are selected, and then matches to these FIKHBs in the UC_STRUCTURES table are retrieved. Likewise, a query requiring a search for the `multiple component InChIs of a single component InChI' would require retrieval of all UC_STRUCTURES with FIKHBs matching the `PARENTS' of a `CHILD' FIKHB corresponding to the query.

The use of the UC_STRUCTURE and UC_FIKHB_HIERARCHY tables working together in this way allows for very fast querying, but retrieves only whole InChIs, and not the separate InChI components of multiple component InChIs, and the separate layers of the single InChIs, required for comparative purposes. For this reason, the retrieved InChIs must be parsed at query time by the application. Although the number of assignments retrieved from a query might be large, the total number of unique Standard InChIs retrieved is often a much smaller number. The speed of processing is thus optimized by ensuring that each unique retrieved InChI is parsed and compared to the Query InChI only once.

### Sources

At the time of writing, UniChem contains over 65 million unique structures, and over 100 million database identifier assignments to these structures, from 22 different sources. The sources of data as well as the content and format required from these sources remains unaltered by the implementation of Connectivity Searching. However, it is important to note some changes to UniChem and the sources that it can access, since its original description [1], which affect the behavior of Connectivity Searching.

Whenever possible, UniChem utilizes Standard InChIs from a source. In the event that Standard InChIs are unavailable, other structural representations (e.g. Molfiles) have been accepted. Recently, UniChem has been modified to accept Standard InChIKeys alone, but will only accept these if a source is unable to provide Standard InChIs or Molfiles, and there are compelling reasons for including the source. These sources within UniChem are classified as `keys only' sources (at the time of writing, only one) and are marked with a `1' in the `keys_only' field in the UC_SOURCE table and on the sources drill-down page on the web interface. If InChIs from another source have InChIKeys matching InChIKeys from such sources, then the InChIs from this second source are assumed to be the correct InChIs for the `keys only' source. Thus, InChIs are only `missing' for the InChIKeys which are unique to the `keys only’ sources. In practice, this is a very small number (currently 1,675 out of a total of nearly 66 million structures in UniChem (i.e. < 0.0026%)).

Querying with, or for, data assigned to `missing’ InChIs using a standard UniChem query (which matches simply on full InChIKey, and does not rely on InChI comparisons) will retrieve matches as normal: The absence of Standard InChIs for some structures originating from `keys_only’ sources makes no difference to the behavior of UniChem under these circumstances. However, running a Connectivity Search query with InChIKeys lacking corresponding InChIs (or querying with the src_compound_ids assigned to these InChIKeys) will, obviously, not retrieve a `Query InChI’ with which UniChem can make comparisons to connectivity-related InChIs. In these circumstances, UniChem cannot run the query, and no data is returned. Likewise, the small number of src_compound_ids assigned to InChIKeys lacking corresponding InChIs will never be retrieved in a result set, because comparisons can only be made when both a Query InChI and a retrieved InChI exist. If a retrieved InChI cannot be obtained for a matching assignment, then the record is skipped.

## Utility and discussion

Connectivity Searching can be carried out using either the web services or a web interface. The web interface is simply a user-friendly front end to the web service, so query construction, search term requirements, and qualifying criteria (Table 1) all have exactly the same meaning for both methods of querying. For full technical details of Connectivity Search, including full descriptions of the criteria that may be applied to run more complex queries, the reader should consult the Connectivity Search Documentation page (located at https://www.ebi.ac.uk/unichem/info/widesearchInfo). For brevity, these details are not reproduced here. Below we describe the use of Connectivity Search via the web interface, examples of its use, and example use-cases for the web-services. Although the description below is largely centered around the web-interface, where small differences exist between the web-interface and the web services, these have been highlighted. Technical details of how the web-services may be employed generally within UniChem (URIs, serialization methods, etc.) are described at https://www.ebi.ac.uk/unichem/info/webservices and are not reproduced here.

**Table 1:** **Summary of search criteria for connectivity searching**

Search criterion	Criterion name	Definition
A	Source	Filter the retrieved results to show only data from a particular src_id (0 = show all sources).
B	Pattern	Define the search pattern (0 = match on FIKHB, 1 = match of Standard InChIKey minus proton flag).
C	Component mapping	Define component mapping. May be set to 1, 2, 3 or 4 (see Table 2).
D	Frequency block	Block sub-queries (where C is set to 1 or 3) for a given single-component InChI on the basis of the frequency of occurrence of this single-component InChI in multiple component InChIs in UniChem.
E	InChI length block	Block sub-queries (where C is set to 1 or 3) for a given a single-component InChI on the basis of the length of the Standard InChI up to the end of the connection layer of the InChI.
F	Labels	Highlight frequently occurring FIKHBs in composite Standard InChIs by adding labels (0 = Add labels, 1 = Do not use labels).
G	Assignment	Assignment status of retrieved data. (0 = only current, 1 = current and obsolete).
H	Structure	Define the data structure of the retrieved data set. [web services only]

Eight search criteria (A-H) may be changed by the user (from their default settings of `0’) in order to refine or qualify the search. Criteria C is defined further in Table 2. Criteria H is only available for use with the web services.

### The user interface

Two search methods are available for Connectivity Search: `cpd_search’ and `key_search’. These methods differ only in the kind of search term that each method accepts. Thus, `key_search’ requires either a full 27 character Standard InChIKey or the 14 character FIKHB, whereas `cpd_search’ requires both a src_compound_id and a src_id. The src_id is required to unambiguously identify the source of the src_compound_id. Within the web-services, the methods are named `cpd_search’ and `key_search’, but in the web-interface the methods are used by selecting either the `src_compound_id’ or the `InChIKey’ radio buttons (respectively).

For the simplest of searches, the user may use all the default settings. Simply hitting the `Submit Query’ button will launch such a query in the web interface. If, however, the user wishes to run a more complex query, they must first select from a series of `Search Criteria’, which serve to qualify and refine the scope of the query, and are described in Table 1. Examples of how these `Search Criteria’ may be used are shown further below. It should be noted that since UniChem is regularly updated, the precise numbers of records retrieved for each example query may vary from those described here, which were accurate at the time of writing.

**Figure 2:**
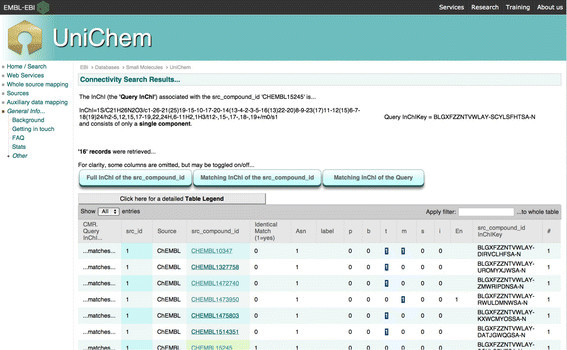
**Connectivity search web interface results page.** The results of a Connectivity Search in the UniChem web interface are shown in a sortable table, with a single matching src_compound_id-to-structure assignment per record. Here are shown the results of a query using src_compound_id CHEMBL15245 (Yohimbine) from the ChEMBL resource. In total, 16 records were retrieved by this query, but for clarity only the first 7 are shown. Comparisons of the individual layers of the Standard InChI are shown (p, b, t, m, s and i), with differences shown with a `1’ (and highlighted), and identical layers shown with a `0’.

Regardless of the `Search Criteria’ used, the results page shows a sortable table of data that contains one record for each of the matching src_compound_id-to-InChI assignments (example of this is shown in Figure 2). The results table also includes information on the differences between the layers of the InChIs retrieved, and those assigned to the query. Thus, matching InChIs which differ in the `i’ (isotopic) layer are shown as a `1’ in the `i’ column, whereas those which do not differ in the `i’ layer are assigned a `0’ in this column. In this way, users may browse their results to identify molecules which share connectivity, but differ in some other aspect of structure. `Labels’ are also included in the results table. These are simple text tags applied to a number of FIKHBs, such as the FIKHB for `HCl’ and other common salts, and are designed to alert the user to the presence of various common salt forms and mixtures. The use of these labels may be avoided by setting criteria F to `1’, and may confer a small performance advantage as a result. The `component mapping’ relationship is also shown as a separate column in this results table. This relationship, defined by criteria C, defines the relationship between the query and retrieved InChIs with respect to whether the matching InChIs are single molecules, or components of a mixture or salt form, and examples of its use are described below.

As a simple example of a Connectivity Search, consider querying with the src_compound_id CHEMBL15245 (Yohimbine) from the ChEMBL resource [12-14]. Querying with this using the non-Connectivity Search (on the UniChem home page) will retrieve only 1 record (ie: itself) from the ChEMBL source. However, with Connectivity Search, using all the default criteria settings (except with criteria `A’ set to `1’, so that only ChEMBL data is retrieved) a total of 16 records are retrieved, as shown in Figure 2. The result set includes molecules such as CHEMBL10347 (Rauwolscine), a stereoisomer of Yohimbine.

If the user wanted to widen this search for stereochemical and isotopic variants of CHEMBL15245, then simply changing criteria C to `4’ would permit all component mapping permutations to be run. This would mean that the query would be widened to include structures that satisfied any of the relationships shown in Table 2. Using these wider settings a total of 21 records are retrieved. In addition to the stereochemical isoforms retrieved before, the new result set now includes salt forms such as CHEMBL537669 (Yohimbine Hydrochloride) and CHEMBL1257131 (Rauwolscine Hydrochloride).

**Table 2:** **Search criterion C is used to define the `Component mapping’ relationship**

Setting	Component mapping relationship.	Description
0	Matches	The Query InChI matches the InChI assigned to the src_compound_id
1	Matches a component of	The Query InChI matches a component of the InChI assigned to this src_compound_id.
2	Has a component which matches	A component of the Query InChI matches the InChI assigned to the src_compound_id
3	Has a component which matches a component of	A component of the Query InChI matches a component of the InChI assigned to src_compound_id
4	-	0-3 simultaneously

Criteria C defines the relationships that will be searched for between the query and retrieved InChIs. C is set to `0’ by default, but may be changed by the user to search with more complex relationships. Setting criterion C to `4’ will run all 4 options (0–3) simultaneously.

A potential problem of changing the component mapping criteria (C) from the default value is that under certain circumstances the number of records retrieved can potentially be vast, and almost certainly not intended or required by the user. Thus, in the example above, suppose we queried with CHEMBL537669 (Yohimbine Hydrochloride) instead. Subqueries using the `Yohimbine’ component of this multi-component query would retrieve alternative salt, stereoisomer and isotopic forms of Yohimbine. However, subqueries with the `Hydrochloride’ component would retrieve all hydrochloride salts of any molecule in UniChem. This could amount to many tens of thousands of different InChIs, if not more. To prevent this, such subqueries are not carried out for components that are present in more than 200 different compounds within UniChem (by default). For some queries where the user is interested in a compound which is quite commonly found as a component of mixtures in UniChem, this setting of 200 may be too low. In these circumstances this sub-query-blocking behavior may be varied using Criteria D and E, but cannot be fully overridden.

Changing other criteria from their defaults can modify the example query above yet again. Thus, setting criteria B to `1’ will result in retrieving only molecules with identical stereochemistry and isotopic composition as the query (although note that if C remains set at `4’, then such molecules within different salts and mixtures will also be retrieved). Likewise, setting criteria G to `1’ in our example above will retrieve the obsolete src_compound_id CHEMBL430347 in addition to all current assignments.

### Use case 1: compound novelty checking using KNIME

The Connectivity Search web services can be called via any programming language or workflow tool, such as Taverna [15,16], KNIME [17,18], or Pipeline Pilot [19]. The latter two in particular, have been increasingly adopted by the computational and medicinal chemistry community, mainly due to their ease of use and the number of chemistry and chemoinformatics extensions available. It is a routine and yet crucial task for medicinal and computational chemists to carefully check whether a compound of interest is truly novel, i.e. it has not been published in scientific literature, claimed in patents, offered by compound vendors, etc. On the other hand, it is quite often that researchers would like to collect as much information about a molecule (or set of molecules) as possible, e.g. whether the compound is synthesizable or purchasable, its reported role in a biological system, a set of references for it, etc. For both the use cases above, we present here a set of KNIME workflows, which facilitate rapid compound novelty checks by using the UniChem Connectivity Search web services. Although UniChem covers the largest public domain patent chemistry corpus, the novelty detection feature is limited at the moment by the lack of exhaustive Markush structure search, which is provided by some commercial products. We will seek future opportunities to address this, as the field progresses.

**Figure 3:**
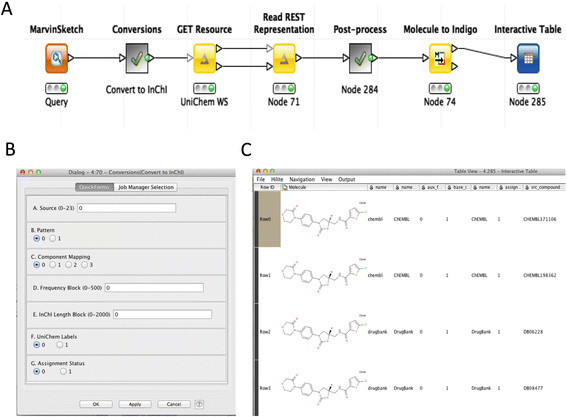
**KNIME workflow for compound novelty checking.** The workflow tool KNIME can be used with Connectivity Search to check for the novelty of a particular compound. **(A)** A summary of the entire workflow, as detailed in the text. **(B)** A KNIME node dialogue allows the users to specify criteria A-H for the Connectivity Search. **(C)** The search hits are returned and converted from InChI strings to molecular images for easier inspection.

A summary of the workflow can be seen in Figure 3A. The user can manually sketch a structure or provide a SMILES or SD file as input. The structures are then converted to InChI keys by one of the KNIME chemoinformatics extensions such as Indigo [20] or RDKit [21]. For each InChI key, the corresponding UniChem web service is called via a GET request, given the additional connectivity search parameters, which can be specified by the user on the node dialogue (Figure 3B). The JSON response is then converted to a KNIME table using the nodes provided by the KREST nodes extension [22]. The output table lists all the sources containing the compound structures of interest along with additional source information, identifiers and hyperlinks to the corresponding sources websites. This information can be further utilized and disseminated in reports, web portals and web pages, etc., as shown in Figure 3C.

### Use case 2: alerting users of one source to alternative molecular forms of a compound in other resources

The Connectivity Search web services can also be called, on the fly, from within the web application of a resource to alert users to alternative molecular forms in other resources. The data retrieved can be straightforwardly parsed and rendered in whatever format the developers of a resource believe is most useful to their users.

**Figure 4:**
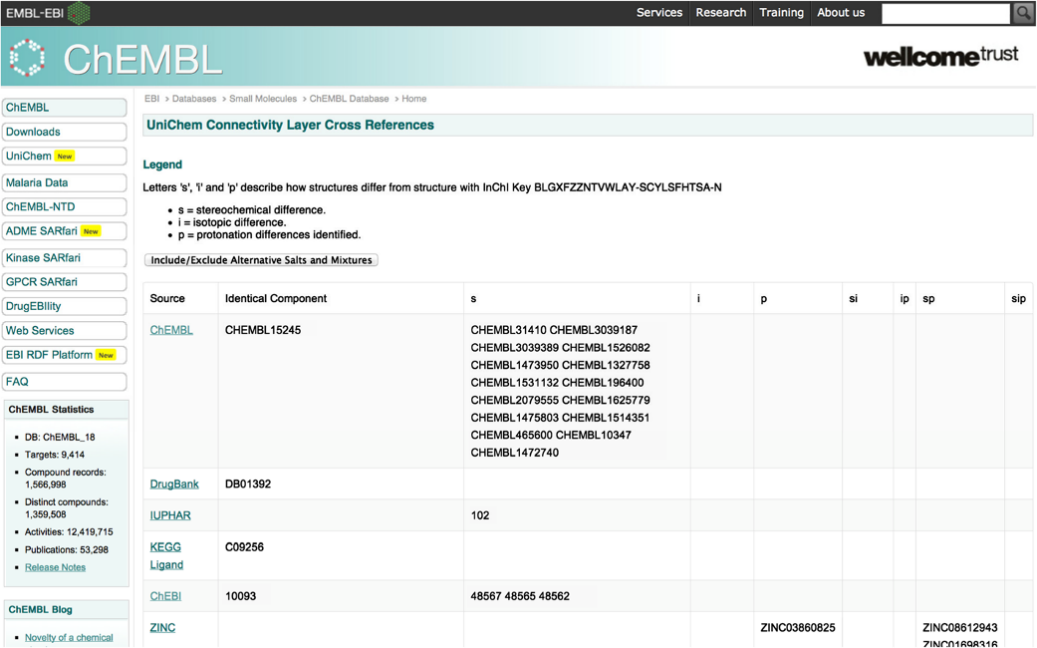
**Using Connectivity Search to alert users of one source to alternative molecular forms of a compound in other resources.** The ChEMBL resource utilizes Connectivity Search to alert users to alternative molecular forms of ChEMBL compounds in other sources. The page shown here, reached from a link from the ChEMBL page for CHEMBL15245, gives full details of all alternative stereoisomers, isotopic variants and salt and mixture forms of CHEMBL15245. In this case, the matching data are clustered by source, although clearly other formats are easily created depending upon the requirements of the users of the resource.

For example, the ChEMBL resource [12]–[14] uses just such a web service query to alert users to alternative molecular forms of ChEMBL compounds that exist elsewhere. Within the ChEMBL web interface each ChEMBL compound has its own dedicated compound page, which summarizes all information relating to that compound in ChEMBL (eg: the number and type of bioassay determinations, etc.). At the foot of such pages, ChEMBL developers have elected to use both normal UniChem queries and Connectivity Search queries to alert their users to the presence of alternative molecular forms of the compound. Thus, a table showing all full Standard InChI matches in other sources is shown to the ChEMBL user, alongside a link to another page, which, if followed, will generate on the fly a more comprehensive set of hyperlinks to alternative salt, stereoisomer and isotopic variants. An example of this page, for CHEMBL15245 (Yohimbine), is shown in Figure 4.

## Conclusion

The InChI was introduced over ten years ago and has become a widely accepted structural representation [2]. Its strength lies in ability to represent a molecule as a string, with increasing levels of structural specification represented in successive layers. This format greatly assists rapid, computationally-based comparison between molecules at different levels of structural definition. The current limitations of InChI are clearly described within the InChI documentation [23]. Thus polymers, Markush structures and non-traditional organic stereochemistry (i.e.: structures other than those containing sp2 and sp3 centers) cannot be represented currently, and larger molecules such as proteins, RNAs and macrocycles can be dealt with, but can generate extremely long InChIs which may be cumbersome to store and manage. It is also noteworthy that the InChI generation software permits users to customize the creation of InChIs according to the level of structural specification required. A disadvantage of this flexibility is that interoperability is compromised, since the same molecule may have a different InChI depending upon the options selected. For this reason, in 2009, the Standard InChI was developed by IUPAC, which is generated using fixed options. The loss of some structural specification as a result of this standardization are documented [23]. UniChem utilizes the Standard InChI and does not accept non-Standard InChIs. The higher levels of structural representation that can only be captured in non-Standard InChIs, and not in Standard InChIs (such as tautomer information, for example), are therefore clearly lost. However, we believe that for the most part the Standard InChI represents structural equivalence in the drug discovery and life sciences context very well, and that the loss of some structural specification as a consequence of standardization is an acceptable trade-off for powerful integration.

However, there are some other limitations of InChI which affect Connectivity Searching, and which should be noted here. Thus Connectivity Searching in UniChem relies entirely upon the ability of InChI software to normalize the connectivity of molecules that may have been drawn in different protonation or charge states. In the vast majority of cases of compounds of biological interest, InChI handles these normalizations extremely well.

For example, for a sodium salt of a carboxylic acid such as p-aminobenzoic acid the InChI software understands that the carboxylic acid has been deprotonated and the InChI is…

InChI = 1S/C7H7NO2.Na/c8-6-3-1-5(2-4-6)7(9)10;/h1-4H,8H2,(H,9,10);/q;+1/p-1

In this case the connectivity layer of the p-aminobenzoate component will match that of p-aminobenzoic acid where the InChI is…

InChI = 1S/C7H7NO2/c8-6-3-1-5(2-4-6)7(9)10/h1-4H,8H2,(H,9,10)

…and so p-aminobenzoic acid will be identified as a component of sodium p-aminobenzoate, and likewise p-aminobenzoic acid will be identified as matching a component of p-aminobenzoic acid.

This works well for most common salts such as carboxylates, phenolates, hydrochlorides and ammonium salts. More details can be found on which salts are represented in their connected or disconnected form elsewhere [23].

However, there are some examples where InChI is not yet able to handle such normalization correctly. Some relatively common acid anions are not recognized as such, and so the relationship between the parent and salt is lost. Sulphonamides and tetrazoles are the most common examples of this but there are others. For example, the Standard InChI for 5-methyl tetrazole is…

InChI = 1S/C2H4N4/c1-2-3-5-6-4-2/h1H3,(H,3,4,5,6)

…whereas the Standard InChI for its sodium salt is…

InChI = 1S/C2H3N4.Na/c1-2-3-5-6-4-2;/h1H3;/q-1;+1

i.e. the InChI software does not know how to protonate the tetrazole and so the Standard InChIs for the tetrazole components of these two compounds are different.

In this case, the two forms do not share the InChI layers that contribute to the FIKHB (ie: the basic (Mobile-H) InChI layer). Clearly, UniChem is not able to correct for these occurrences, as it relies solely on the InChI software to create connection keys. Users should therefore be aware of these shortcomings, as in some cases this can explain the absence of connectivity matches that may have otherwise been expected.

The original aims of UniChem were to provide a simple, fast, freely available, and low-maintenance mapping service for creating hyperlinks between chemistry data objects in different Internet resources. The benefits of this model have been discussed previously [1]. The current work sought to build on this model to provide a mapping service with the same benefits as before, but where the end user or developer is able to define for themselves more flexibly the criteria for defining molecular equivalence between interlinked resources. This flexibility in the definition of molecular equivalence is important because users from different domains of science are likely to have different views on which molecules they consider can be normalized to a single entity for the purposes of their analyses, and which differences between molecules should be highlighted and annotated. By identifying these related molecules, and defining the differences between them, Connectivity Search provides a tool that can be tailored by the developers of each resource differently to annotate related molecules in ways which suit their user base.

Connectivity Searching may also have an important role to play where the correct depiction of a molecule is under debate. Such debates are common [24,25] for example, and since we suspect that the growth of chemistry databases will continue to outstrip the resources to curate them, more automated mechanisms for identifying incorrectly represented molecules would be useful. Also, because incorrectly curated molecules are always likely to take some significant time to fix, it is important that in the meantime users are not denied the opportunity to link between these disputed versions, and perhaps to decide for themselves which of them are correct. Because atom connectivity is less commonly disputed in these debates, Connectivity Searching provides a mechanism for easily identifying and creating links between these molecules.

## Availability and requirements

UniChem may be accessed at the following URL: https://www.ebi.ac.uk/unichem/ and data is freely available from this site, via the web interface or web services, under a Creative Commons Attribution (CC-BY) license. Connectivity Searching can specifically be accessed at the following URL: https://www.ebi.ac.uk/unichem/widesearch/widesearch and up to date documentation accessed at this URL: https://www.ebi.ac.uk/unichem/info/widesearchInfo.

Both exact and Connectivity Search UniChem KNIME example workflows are freely available for download from ftp://ftp.ebi.ac.uk/pub/databases/chembl/UniChem/KNIME. Moreover, they are also available, along with several other ChEMBL-related workflow examples, in the KNIME EXAMPLES public server, which is accessible directly via the KNIME desktop.

## Authors' contributions

JC designed and implemented modifications to the UniChem database, loaders, supporting code, web interface and RESTful web services, and is currently responsible for all content. MD carried out deployment of the service within the wider EMBL-EBI infrastructure. JC, AG, JPO, AH, MD all contributed to early discussions on the conception and design of the project. AH tested and critically evaluated the web interface and web services, and explored and defined the use-cases and limitations of the service. GP developed the accompanying KNIME protocols and critically evaluated the web services. All authors read and approved the manuscript.

## Authors’ original submitted files for images

Below are the links to the authors’ original submitted files for images. Authors’ original file for figure 1 Authors’ original file for figure 2 Authors’ original file for figure 3 Authors’ original file for figure 4

## Abbreviations

FIKHB: First InChIKey hash block
EMBL: European molecular biology laboratory
EBI: European bioinformatics institute
