# Supplementary figures and images for: UniChem: extension of InChI-based compound mapping to salt, connectivity and stereochemistry layers
Source: J Cheminform. 2014 Sep 4;6:43. doi: 10.1186/s13321-014-0043-5 (PMC4158273; doi:10.1186/s13321-014-0043-5)

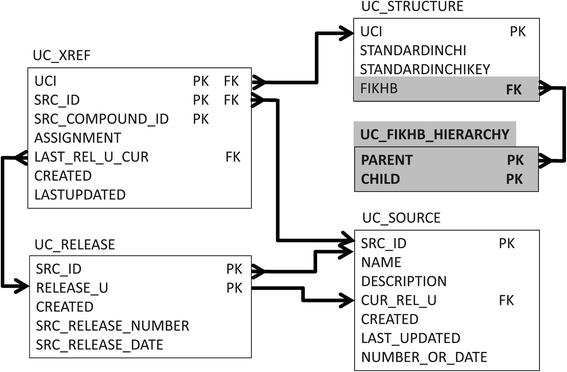

Supplement: Supplementary file 1 — Authors’ original file for figure 1 [file 13321_2014_43_MOESM1_ESM.gif]

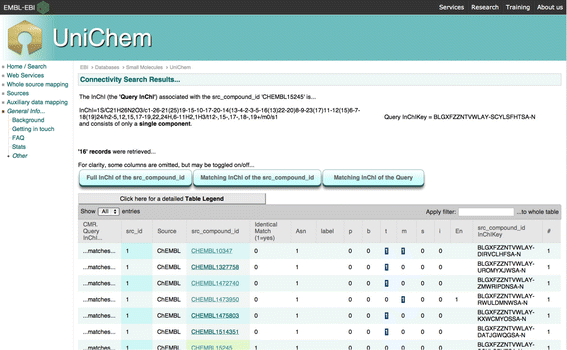

Supplement: Supplementary file 2 — Authors’ original file for figure 2 [file 13321_2014_43_MOESM2_ESM.gif]

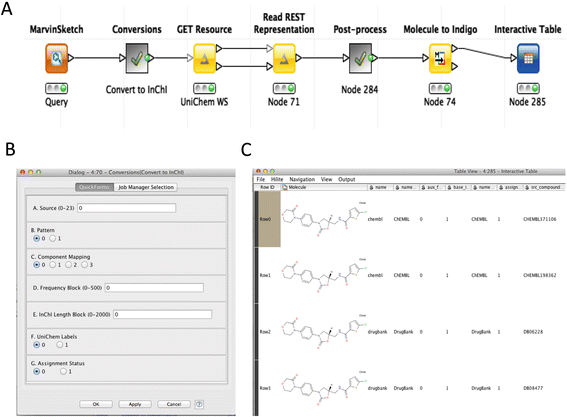

Supplement: Supplementary file 3 — Authors’ original file for figure 3 [file 13321_2014_43_MOESM3_ESM.gif]

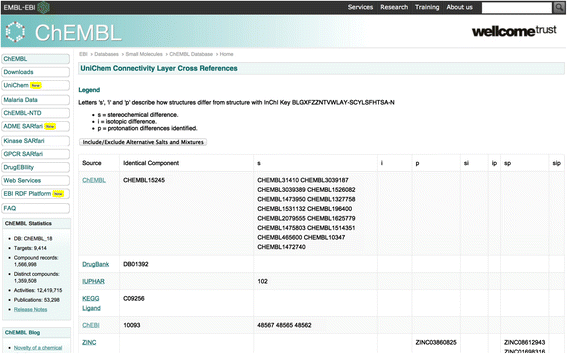

Supplement: Supplementary file 4 — Authors’ original file for figure 4 [file 13321_2014_43_MOESM4_ESM.gif]
